# Self-concealment predicts use of secrecy and attitude toward secrecy, not subjective ability to keep secrets

**DOI:** 10.1007/s12144-025-07738-7

**Published:** 2025-04-02

**Authors:** Laura Blomqvist, Ildikó Éva Csizmazia, Ruth Van der Hallen

**Affiliations:** Clinical Psychology, Department of Psychology, Education & Child Studies, Erasmus University Rotterdam, Rotterdam, 3062 PA The Netherlands

**Keywords:** Secrecy, Secret, Self-concealment, Ability, Attitude, Individual differences

## Abstract

Self-concealment and secrecy, although conceptually distinct, are often conflated or inadequately distinguished in existing literature. In this correlational study, we aimed to elucidate the relationship between self-concealment and three dimensions of secrecy: the use of secrecy, the ability to keep secrets, and attitude toward secrecy. The sample consisted of 220 individuals (76% identified as female), between the ages of 18 to 78 (*M* = 24.27, *SD* = 8.88). Participants completed an online survey which included the Self-Concealment Scale (SCS), the Common Secrecy Questionnaire (CSQ) and the newly developed Secrecy Ability Scale (SAB) and Secrecy Attitude Scale (SAT). Data were screened to mitigate both positive and negative response biases. The results revealed that high self-concealers tend to keep more secrets and have a more positive attitude toward secrecy compared to low self-concealers. No significant group difference emerged concerning the subjective ability to keep secrets. Limitations and future implications of the findings are discussed.

Self-concealment and secrecy, although conceptually distinct, are often conflated or inadequately distinguished in existing literature. Secrecy, a prevalent phenomenon in our everyday lives, is typically defined as the intention to keep some personal information secret from one or multiple people (Slepian et al., 2017). Most of us keep at least one secret at any given time, whether it involves concealing positive information, such as a surprise party, or negative information, such as instances of misconduct. Self-concealment, on the other hand, represents a trait characterized by the tendency to keep negatively valenced personal information, such as intimate feelings and thoughts, hidden from others (Larson & Chastain, 1990). It is an enduring characteristic that influences how individuals interact with others and what is or is not shared with others. Understanding secrecy and self-concealment in a more comprehensive manner as well as how they relate to each other is important as these constructs significantly shape our everyday social interactions and can have a substantial influence on our mental and physical well-being (Bedrov et al., 2021; Davis, 2023; Slepian et al., 2017). That is, individuals who tend to keep secrets and those characterized as high self-concealers are often suggested to struggle with various negative health problems. For instance, secrecy has been associated with elevated stress, diminished health, and increased physical illness risk (Cole et al., 1996; Finkenauer & Rime, 1998; Pennebaker et al., 1988). Similarly, self-concealment correlates with heightened anxiety, depression, suicidal behaviors, and chronic pain (Friedlander et al., 2012; Ichiyama et al., 1993; Larson & Chastain, 1990). The concepts of secrecy and self-concealment have been of interest for a long time (Bellman, 1979; Davidson, 1893; Rowland, 1985) but the way they are defined and understood has evolved over the years, leaving room for ongoing conceptual refinement and clarity.

The conceptualization of secrecy has evolved significantly over the years. Initially, secrecy was defined as “intentional concealment” (Bok, 1989), mirrored by scholars referring to secrecy as the deliberate hiding of information from at least one other person (Kelly, 2002), as active inhibition of disclosure (Pennebaker, 1988), or as intentional deception via an act of omission (Lane & Wegner, 1995). Later on, Wegner and Lane (2004) expanded on the existing conceptualization of secrecy in that secrecy does not require the active involvement of a second party but can be done in isolation. In other words, an individual can keep a secret even in the absence of another person. Nowadays, secrecy is understood as a broad concept of not only actively and intentionally keeping secrets in social interactions, but also the experience of having secrets outside of active concealment (Slepian, 2022). According to Slepian, the essence of secrecy begins the moment a decision or intention is formed to withhold or hide information from someone, regardless of whether that person is physically present (Slepian et al., 2017). In fact, a person keeping a secret might never find themselves in a situation requiring active concealment, i.e., having to take deliberate actions to prevent others from uncovering the secret, such as avoiding specific topics of conversation or providing misleading information. However, the intent to keep the secret remains even in absence of active concealment, and thus the secret exists. This is an important addition to our conceptualization of secrecy as previous definitions have largely overlooked the importance of understanding secrecy outside of the realm of active concealment.

The conceptualization of self-concealment has also evolved over the last few decades. Although references to self-concealment began to appear in academic papers as early as the 1960s (Bugental et al., 1968; Hurley & Hurley, 1969), it was only formally defined and differentiated from self-disclosure by Larson and Chastain in 1990. Larson and Chastain (1990 p. 439) defined self-concealment as a “predisposition to actively conceal from others personal information that one perceives as distressing or negative.” According to their definition, self-concealed personal information is “a) a subset of private personal information; b) consciously accessible to the individual, as distinguished from “unconscious secrets” or secrets from oneself resulting from repression or denial; and c) actively kept from the awareness of others” (Larson & Chastain, 1990 p. 440). Self-concealment has also been referred to as “inhibited personality” (Uysal et al., 2010 p. 188) and considered a maladaptive mood regulation process (Masuda et al., 2011) highlighting the potentially negative impact of a tendency to self-conceal on an individual’s life. While the exact definition of self-concealment remains a topic of debate, Larson and Chastain’s view currently stands as the most prominent in the literature.

Importantly, both secrecy and self-concealment have been associated with adverse health outcomes. For instance, secrecy has been consistently linked to negative health consequences, including but not limited to elevated stress levels, an increased risk of cancer and infectious diseases, heightened illness scores, diminished overall health and well-being (Cole et al., 1996; Finkenauer & Rime, 1998; Pennebaker et al., 1988; Quinn & Chaudoir, 2009; Slepian et al., 2017). Similarly, self-concealment has been associated with compromised physical and mental health, including chronic and acute pain (Larson & Chastain, 1990; Uysal & Lu, 2011), suicidal behaviors, depression, negative self-esteem and increased anxiety (Friedlander et al., 2012; Ichiyama et al., 1993; Larson & Chastain, 1990). Recent research, however, challenges the conventional understanding that the mere act of keeping a secret is the primary culprit behind these detrimental health effects. Instead, it appears that the trait-level predisposition to self-conceal may exert a more substantial impact on an individual’s mental and physical well-being (Kelly & Yip, 2006). This idea gains support from studies that have found symptom reduction in outpatient therapy when self-concealment was controlled for, suggesting that, in some therapeutic contexts, concealing certain aspects of oneself from a therapist might yield positive benefits (Kelly, 1998). Additionally, a study by Maas and colleagues (2019) has indicated that secret keeping no longer had a negative effect on quality of life when controlling for self-concealment, supporting the view that it may well be the tendency to self-conceal rather than the act of keeping a secret that contributes to the development of negative consequences to the individual. This underscores the possibility that it is the tendency to self-conceal, rather than simply keeping a secret, that contributes to the development of adverse consequences for an individual’s well-being. However, as previous research has rarely differentiated clearly between the concepts of secrecy and self-concealment, it remains challenging to draw separate conclusions about their respective effects on mental or physical health (Kelly & Yip, 2006).

In fact, the academic literature tends to lack a clear distinction between the concepts of secrecy and self-concealment, and both terms are sometimes used interchangeably. For instance, when reading the title of a paper by Hartman and colleagues (2015 p. 1), “Direct and indirect links between parenting styles, self-concealment (secrets), impaired control over drinking and alcohol-related outcomes” the lack of differentiation between the two concepts becomes evident immediately. Moreover, in an article by Maas and colleagues (2019 p. 251), self-concealment is referred to as “secrecy as a trait” which would suggest that self-concealment is considered a component or aspect of secrecy rather than a distinct concept. In addition, the way both concepts are measured, can sometimes contribute to confusion. While distinct measures exist for either concept, such as the Common Secrets Questionnaire to measure secrecy (Slepian et al., 2017) and the Self-Concealment Scale to measure self-concealment (Larson & Chastain, 2016), they are occasionally interchanged in research. For instance, in a recent paper by Musetti and colleagues (2019), secrecy was operationalized using an adapted version of the Self-Concealment Scale. This mixing of measures can understandably lead to confusion regarding the relationship and level of interconnectedness between secrecy and self-concealment.

Nevertheless, scholars have undertaken significant efforts to shed light on the relationship between secrecy and self-concealment. For instance, following a meta-analytic investigation that included 137 studies that used the Self-Concealment Scale, a working model of self-concealment was formulated. Among its key findings, the study revealed that self-concealment is indeed positively associated with secret-keeping (*r* = .35), yet that they are distinct concepts (Larson et al., 2015). This aligns with previous work which tends to report moderate correlations between secrecy and self-concealment (Kelly, 1998; Kelly & Yip, 2006; Maas et al., 2012, 2019), indicating that high self-concealers typically also tend to keep more secrets (Davis, 2023; Kelly & Yip, 2006; Maas et al., 2012, 2019). Importantly, this association is primarily observed for secrets related to the self that individuals perceive as negative, rather than all types of secrets (Wismeijer, 2011).

One potential hindrance in elucidating the relationship between self-concealment and secrecy, may be the fact that secrecy is predominantly considered in terms of the secrets we keep. While the literature acknowledges that secrecy is an ability that develops with age (Bedrov et al., 2021; Meares & Orlay, 1988; Misch et al., 2016; Peskin & Ardino, 2003) and that individuals cultivate attitudes toward secrecy (Black, 1988; Myrick, 2020; Szajnberg, 1989), these aspects are merely considered in theoretical rather than empirical research on secrecy. Yet, one’s ability and one’s attitude are typically considered important determinants of behaviors more in general. For example, in the context of suicide research, it has been established that for an individual to carry out suicidal behavior (i.e., attempted or completed suicide), they need to have a certain ability to self-harm (Van Orden et al., 2005) and a more favorable attitude toward suicide has been linked to both increased suicidal ideation and suicidal behavior (Diekstra & Kerkhof, 1988; Kodaka et al., 2011). Similarly, research into lying behavior has demonstrated that factors such as one’s ability to lie (Michels et al., 2020; Vrij et al., 2010), and one’s attitude toward lying (Buta et al., 2020; Curtis, 2021) significantly influence a person’s tendency to lie. Considering these parallels, it stands to reason that an individual’s subjective ability to keep secrets and their attitude toward secrecy are pertinent factors in trying to understand the concept of secrecy and how it may relate to self-concealment.

The current study aimed to deepen our understanding of the intricate relationship between self-concealment and secrecy. Specifically, this study aimed to investigate whether individuals characterized as high and low self-concealers differ in their use of secrecy, their ability to keep secrets, and their attitude toward secrecy. In our rapidly evolving digital age, characterized by social phenomena such as the MeToo movement gaining traction on various social media platforms (Siuta et al., 2023), individuals are increasingly inclined to disclose information they might have previously kept secret. The widespread adoption of social media channels has provided a platform for individuals to anonymously share personal experiences that they might have hesitated to reveal in the past (Sharon & John, 2018). Given this shifting societal landscape, it is imperative to gain a deeper understanding of the traits that influence an individual’s decision to disclose or keep personal information secret. Notably, this study represents this first study to investigate how self-concealment is associated with frequency, ability, and attitude toward secrecy. Based on the reviewed literature, we hypothesized that high self-concealment is positively correlated with all three secrecy outcomes (H1), meaning a higher use of secrecy (H2), an enhanced ability for secrecy (H3) and a more favorable attitude toward secrecy (H4). Moreover, we anticipate that the observed differences between high and low self-concealers will be most pronounced concerning H2, while the effect will be relatively weaker concerning H4. Altogether, this research aims to contribute valuable insights into the intricate relationship between self-concealment and secrecy, ultimately fostering a more nuanced understanding of individuals’ choices to disclose or keep secret certain information and how that is related to mental and physical health.

## Methods

### Participants

A total of 570 individuals responded to our invitation, of which 553 provided informed consent and filled in the survey. Prior to data analysis, respondents showing a positive and/or negative response bias were excluded from the dataset (*n* = 143, see Materials for more details). Following the initial exclusion, respondents who scored within the lowest quartile (Q1) and the highest quartile (Q4) on the Self-Concealment Scale (see Materials) were included in the final sample. This resulted in a final sample of 220 individuals (75.9% identified as female). Ages ranged from 18 to 78, with a mean of 24.27 (*SD* = 8.88). Respondents reported their sexual orientation as straight (69.7%), bisexual (18.6%), gay/lesbian (5.9%), pansexual (4.1%), asexual (0.9%), other (1.8%), and prefer not to say (0.9%). A significant proportion of the final sample consisted of Erasmus University Rotterdam students who participated in the study for course credit (65.5%). Respondents were geographically diverse, representing the six continents of Europe, Africa, Asia, Eurasia, North America, and South America, with most participants identifying as Dutch (41.1%), Greek (11.4%), and German (7.7%).

### Procedure

The present study is part of a larger research project that explores the concept of secrecy in conjunction with various other constructs. Ethical approval was granted by the Erasmus School of Social and Behavioral Sciences (ESSB) Research Ethics Review Committee prior to data collection. Participation in the study was voluntary and individuals aged 18 and above were eligible to take part. Erasmus University Rotterdam students were offered course credit as an incentive for their involvement, while non-students had the opportunity to enter a draft lottery for a chance to win one of ten BOL.com vouchers worth 10 euros each (1/10 chance to win). Respondents were recruited using the Erasmus University recruitment platform (ERAS) as well as various social media platforms, including Instagram, Facebook, and WhatsApp. Recruitment posts provided a concise overview of the survey’s main objectives and anticipated duration (approximately 30 min), along with a hyperlink to access the Qualtrics survey. The survey included some general demographic questions such as nationality, age, sexual orientation, and gender. Subsequently, respondents completed several scales, including the Self-Concealment Scale (SCS), Common Secrets Questionnaire (CSQ), Secrecy Ability Scale (SAB), Secrecy Attitude Scale (SAT), Supernormality Scale (SS-R), and Brief Symptom Inventory (BSI-18), which was supplemented with four items from the Wildman Symptom Checklist.

### Materials

The Self-concealment Scale (SCS; Larson & Chastain, 2016) was used to measure individuals’ predisposition to conceal personal information from others. The SCS comprises 10 items that gauge one’s tendency to actively conceal personal information that one considers distressing or negative in nature. An example item is: “My secrets are too embarrassing to share with others”. Respondents rated each item on a 5-point Likert scale, ranging from 1 (strongly disagree) to 5 (strongly agree). Total SCS scores can vary from 10 to 50, with higher total scores indicating a greater tendency to self-conceal (Larson & Chastain, 1990). The SCS is characterized by high internal consistency (.83, Larson & Chastain, 1990; .91 for the current sample) and good test-retest reliability (.81, Larson & Chastain, 1990).

The Common Secrets Questionnaire (CSQ; Slepian et al., 2017) was used to measure the number or frequency of secrets individuals have or have had. The CSQ comprises 38 items, covering various themes of secrets individuals may have experienced. An example item is: “Had romantic desires about someone (while being single)”. Respondents were instructed to select the most appropriate response option from: “I have had this experience, and keep it secret from everyone”, “I have had this experience, and keep it secret from some people”, “I have had this experience, and once kept it a secret, but it is not a secret anymore”, “I have had this experience, but I have never kept it a secret”, “I have never had this experience”. A ratio score was computed by dividing the number of experiences a person kept or is keeping a secret by the total number of experiences they have had. For instance, an individual who endorsed experiencing 35 out of the 38 items but only kept 25 of them as secrets would receive a ratio score of 24/35 = 0.71. CSQ ratio scores can range from 0 to 1, with higher scores indicating a greater propensity for using secrecy. Cronbach’s alpha level for the current sample was .75.

The Secrecy Ability Scale (SAB; Van der Hallen & Csizmazia, 2023) was used to assess individuals’ ability to keep secrets. The scale consists of three general statements (1–2) regarding one’s capacity to maintain secrecy and seven more specific statements (3–9) relating to various aspects of secret-keeping. An example item is: “I can easily keep something a secret even from people who I love”. Respondents rated each item on a 5-point Likert scale, ranging from 1 (strongly disagree) to 5 (strongly agree). Average scores, ranging from 1 to 5, are calculated, with higher scores indicating a greater ability to keep secrets. Cronbach’s alpha level for the current sample was .88.

The Secrecy Attitude Scale (SAB; Van der Hallen & Csizmazia, 2023) was used to measure individuals’ attitudes toward secrecy. The scale comprises 10 items that assess the affective aspect of attitude (1–3), participants’ behavioral tendencies when keeping a secret (4–7), and the cognitive aspects of one’s attitude toward secrecy (8–10). An example item is: “It is immoral to keep secrets”. Respondents rated each item on a 5-point Likert scale, ranging from 1 (strongly disagree) to 5 (strongly agree). Average scores, ranging from 1 to 5, are calculated, with higher scores indicating a positive attitude toward secrecy. Cronbach’s alpha level for the current sample was .78.

The Supernormality Scale Revised (SS-R; Cima et al., 2008) was used to detect the presence of a positive response bias. Comprising 50 items, the scale incorporates 16 so-called “bogus items” strategically included to obscure the true purpose of the scale. The SS-R assesses the occurrence of symptoms commonly experienced by individuals at some point in their lives. An example item is: “I sometimes imagine being in a different place”. Respondents indicated the frequency with which they were troubled by these symptoms on a 4-point Likert scale, ranging from 1 (never) to 4 (always). SS-R total scores can range from 34 to 136 (bogus items excluded). Given that the symptoms assessed by the scale are prevalent in the general population, respondents scoring 60 or lower are considered to show a positive response bias. The SS-R is characterized by good internal consistency (.88; Cima et al., 2008; .86 for the current sample).

The Brief Symptom Inventory (BSI-18; Derogatis, 2001) is a shortened version of the 53-item BSI, developed to assess the presence of psychological symptoms in the general population. Four items from the Wildman Symptom Checklist (WSC; Wildman & Wildman, 1999) that address non-credible symptoms were interspersed among the BSI-18 items. The selected items are used to identify symptom overreporting or so-called negative response bias (Merckelbach et al., 2008, 2014). Original items of the BSI-18 are discarded; instead, the non-credible symptom items are rated on a 5-point Likert scale, ranging from 0 (not at all) to 4 (all the time). A symptom overreporting total score is calculated, ranging from 0 to 16, with scores of above 4 considered indicative of negative response bias (Merckelbach et al., 2008, 2014).

### Data-analysis

Prior to the data analysis, the dataset was screened for outliers as well as response bias. Outliers were examined using Mahalanobis distance, Cook’s distance, and boxplot graphs. No outliers were identified. Although Box’s test indicated heterogeneity of the covariance matrices, given the sample sizes in the groups are equal, Hotelling’s T2 is deemed robust (Hakstian et al., 1979). Response bias was assessed using the SS-R and WSC items, resulting in the exclusion of 143 (25.9%) respondents. Furthermore, due to the low number of respondents identifying as “non-binary” (10), “other” (1), or “prefer not to say” (3), these participants were excluded from the analysis. No missing data was present in the sample.

To investigate the relationship between self-concealment and secrecy, a MANCOVA was conducted with self-concealment as independent variable and the use of secrecy, the ability to keep secrets, and attitude toward secrecy as dependent variables. Gender was included as a covariate. Self-concealment was treated as a binary variable with respondents scoring in the lowest quartile (Q1) categorized as low self-concealers (coded as 0) and those scoring in the highest quartile (Q4) categorized as high self-concealers (coded as 1). Respondents scoring in Q2 and Q3 on the SCS were excluded from further analysis. Gender was treated as a binary variable, categorizing individuals as either female (0) or male (1). The category “female” encompassed both cisgender females and transgender females, while the category “male” included both cisgender males and transgender males. An alpha level of .05 was used for all statistical analyses. All statistical analyses were conducted using SPSS, version 29.0.

## Results

### Descriptives

The descriptive statistics indicate that respondents, on average, tended to keep 76% (*SD* = 18.67) of their experiences a secret, as measured by the CSQ. Common experiences that were kept a secret included lying to someone, harboring romantic desires about someone while being single, and withholding certain family details. Additionally, respondents overall reported a high level of ability to keep secrets and held a moderately positive attitude toward secrecy. Table 1 provides a comprehensive overview of the descriptive statistics and Pearson’s correlation coefficients.

**Table 1:** Descriptive statistics and Pearson’s correlation coefficients of the main variables

	Total (*n* = 220)		Female (*n* = 167)		Male (*n* = 53)		Correlations			
Variable	*M (SD)*	Range	*M (SD)*	Range	*M (SD)*	Range	1	2	3	4
1. SCS	28.72 (10.95)	10–50	27.72 (10.94)	10–48	31.87 (10.43)	10–50	-	.42^**^	.08	.38^***^
2. CSQ	0.76 (0.19)	0–1	0.76 (0.19)	0–1	0.76 (0.18)	0.22 − 1		-	.16^*^	.26^***^
3. SAB	3.51 (0.87)	1.3–5.0	3.45 (0.85)	1.4–5.0	3.71 (0.89)	1.3–5.0			-	.32^**^
4. SAT	3.50 (0.61)	1.2–5.0	3.46 (0.62)	1.2–5.0	3.62 (0.56)	2.2–4.6				-

Note. SCS = Self-Concealment Scale; CSQ = Common Secrets Questionnaire; SAB = Secrecy Ability Scale; SAT = Secrecy Attitude Scale. * *p* < .05 (two-tailed). **p < .01 (two-tailed)

### Main analyses

A multivariate analysis of covariance (MANCOVA) was conducted to investigate self-concealment in relation to the use of secrecy (CSQ), the ability to keep secrets (SAB) and individuals’ attitude toward secrecy (SAT), while controlling for gender. The MANCOVA yielded a significant multivariate effect of the level of self-concealment on the various secrecy outcome measures, Hotelling’s *T*^2^ = 0.26 *F*(3, 215) = 18.96, *p* < .001, *η*^2^partial = 0.21, thus supporting hypothesis (1) Subsequent univariate ANCOVA’s revealed a significant univariate effect of the level of self-concealment on use of secrecy and attitude toward secrecy but not ability to keep secrets. More specifically, high self-concealers reported a higher use of secrecy (*M* = 0.83, *SD* = 0.14*)* compared to low self-concealers (*M* = 0.69, *SD* = 0.20*)*, *F*(1, 217) = 35.18, *p* < .001, *η*^2^partial = .14, supporting hypothesis (2) In addition, high self-concealers reported a more positive attitude toward secrecy (*M* = 3.70, *SD* = 0.54*)* compared to low self-concealers (*M* = 3.30, *SD* = 0.61), *F*(1, 217) = 24.38, *p* < .001, *η*^2^partial = .10, supporting hypothesis 4. Surprisingly, there was no significant group difference in self-reported ability to keep secrets between high self-concealers (*M* = 3.53, *SD* = 0.87) and low self-concealers (*M* = 3.50, *SD* = 0.86), *F*(1, 217) = 0.00, *p* = .10, contrary to hypothesis 3.

## Discussion

The current study aimed to provide a comprehensive understanding of the intricate relationship between self-concealment and secrecy. The findings of this study align with previous research, confirming a moderate correlation between self-concealment and secrecy (Kelly & Yip, 2006; Maas et al., 2019). High self-concealers were found to keep more secrets, suggesting that individuals who tend to hide distressing or negative personal information are more inclined to keep secrets. Furthermore, high self-concealers exhibited a more favorable attitude toward secrecy, reinforcing the idea that their inclination to habitually conceal negative personal information contributes to a positive attitude toward secrecy. Interestingly, no significant relationship was revealed between individual’s level of self-concealment and their ability to keep secrets. Taken together, these findings contribute to our understanding of the interplay between self-concealment and secrecy.

With regard to the use of secrecy between high and low self-concealers, the results of this study align with previous findings (Kelly & Yip, 2006; Larson et al., 2015; Maas et al., 2012), providing support for our hypothesis. Specifically, high self-concealers were found to report a greater use of secrecy compared to low self-concealers. This outcome resonates with the working model of self-concealment (Larson et al., 2015) which posits that self-concealment tendencies manifest in the form of secret-keeping behavior. An interesting observation is that, while previous research has not found an association between self-concealment and secret keeping in relation to rule violations and taboo topics (Wismeijer, 2011), a substantial portion of the CSQ items pertains to rule violations and taboo topics (approximately 20–30%). Wismeijer suggests that individuals with a greater tendency to self-conceal, exhibit traits of inhibition, which should theoretically result in fewer rule violations (2011). Nevertheless, the current study found a significant relationship between one’s level of self-concealment and the extent to which one is likely to keep their experiences as secret. One possible explanation, contrary to prevailing views in the field, is that high self-concealers tend to engage in secret-keeping across various life domains, rather than exclusively in relation to negatively valenced personal information. Although prior research suggests that self-concealment “is indeed primarily related to a subset of privately held convictions of negative aspects of oneself as opposed to keeping many secrets in general” (Wismeijer, 2011 p. 1042), it is important to note that there is a lack of consensus in the field regarding the specific scale that should be used to measure said secret-keeping behaviors. Unlike self-concealment research, which predominantly employs the Self-Concealment Scale (Larson & Chastain, 2016), secrecy research encompasses a wide range of scales with varying item counts and item contents, making direct comparisons between studies challenging (Kelly & Yip, 2006; Slepian et al., 2017).

Regarding the relationship between self-concealment and attitude toward secrecy, the current study provides novel insights by confirming, as hypothesized, that high self-concealers tend to hold a more positive attitude toward secrecy compared to low self-concealers. Prior research has suggested that one’s attitude toward secrecy can vary depending on whether the focus is on the secret-keeper’s attitude or the attitude of the person from whom the secret is being kept (Finkenauer & Hazam, 2000). In line with this perspective, the current study found high self-concealers, who were found to be more likely to engage in secret-keeping behavior, to also exhibit a more positive attitude toward secrecy.

An additional lens through which to interpret this result is the cognitive dissonance theory, proposed by Festinger (1957). According to the cognitive dissonance theory, individuals experience psychological discomfort when their beliefs, attitudes, and behaviors are not properly aligned with each other (Harmon-Jones & Mills, 2019). To alleviate this dissonance, the theory suggests, individuals are motivated to either adjust their beliefs or behavior or to seek out information that reinforces their existing beliefs to maintain internal consistency. As such, it is plausible that high self-concealers, who tend to keep more secrets, actively seek, and focus on information that confirms their pre-existing beliefs that secrecy is not inherently bad and thus, may uphold a more positive attitude toward it. In essence, high self-concealers may engage in a cognitive process that allows them to rationalize their secret-keeping behavior and view it in a positive light, even if they experience feelings of guilt or shame about it (Slepian et al., 2020).

Interestingly, this study did not find a significant difference between high and low self-concealers in their self-reported ability to keep secrets. Although no previous research has investigated the relationship between self-concealment and the ability to keep secrets, it defied the initial hypothesis that high self-concealers would report a superior ability in keeping secrets. This hypothesis was grounded in the idea that one’s ability to keep secrets is honed through practice and is influenced by one’s confidence in keeping secrets (DePaulo, 1992). Given that high self-concealers have been shown to keep more secrets and consequently have had more practice in secret keeping, it seemed reasonable to presume they would also report a greater ability to do so. One potential explanation as to why the present study did not find a significant group differences in secret-keeping ability, is the potential high baseline level of secret-keeping proficiency among most individuals. This idea is supported by the narrower range of the ability scores (1.3–5.0) as observed within the survey. Additionally, not a single respondent reported not being able to keep secrets altogether. In fact, the ability to keep secrets is considered a hallmark of normal adult functioning (Kelly, 2002), suggesting that most adults possess a baseline capacity for secret-keeping. Since the current sample exclusively comprised adult respondents, it is plausible that all respondents believed they possessed at least some ability to keep secrets. Furthermore, previous research has indicated that individuals tend to overestimate their own abilities, particularly when their competence in a particular task is in fact relatively low (Bench et al., 2015; Dunning et al., 2003; Mahmood, 2016). As Dunning (2011 p. 280) noted, “decades of research suggest that the impressions people have of their skill are only weakly to modestly correlated with objective performance.” In other words, it is possible that respondents in the current study overestimated their subjective ability to keep secrets, contributing to the non-significant finding. Lastly, the relatively young age of the participants in this study could have also played a role in the non-significant result. Previous research has found that younger individuals tend to score higher on self-concealment compared to older individuals (Friedlander et al., 2012; Masuda et al., 2017). Given that the mean age of the current sample was approximately 25, it is plausible that participants in both groups demonstrated high secret-keeping ability, likely due to their recent and frequent experiences with secret-keeping behaviors.

The current study presents two significant strengths that warrant recognition. First and foremost, our study adopted a comprehensive approach by considering three distinct facets of secrecy, in contrast to the common practice of focusing solely on the type or quantity of secrets individuals keep. This multifaceted perspective enriches our understanding of the intricate relationship between secrecy and self-concealment. By examining how self-concealment interplays with the use of secrecy, the ability to keep secrets, and one’s attitude toward secrecy, we deepen our insights into how self-concealment influences various aspects of secrecy and the ways in which they are interconnected. Secondly, our study included a relatively large sample size which, coupled with the exclusion of respondents exhibiting positive or negative response biases, strengthens the reliability of our results (Cima et al., 2008; Merckelbach et al., 2014). In summary, these strengths enhance the validity of our findings and provide a robust foundation for future research in this domain.

Nevertheless, it is important to recognize the limitations of this study and highlight potential avenues for future research. Firstly, the present study was correlational in nature and therefore, no causational relationships can be inferred from the results. As such, it is not possible to assert that self-concealment causes certain secrecy behaviors, as the relationship could just as well be reversed, or both could be influencing each other. Longitudinal research is necessary to gain more insight into the causal pathways involved. A second limitation concerns the stability of self-concealment as a personality trait (Larson et al., 2015). Some research has indicated that self-concealment might operate differently throughout the lifespan (Friedlander et al., 2012) which restricts the generalizability of the results beyond the present time point. Future studies could adopt a longitudinal approach to investigate how the relationship between self-concealment and various aspects of secrecy develop over time. This could include exploring, for instance, whether self-concealers’ attitude toward secrecy or their ability to keep secrets vary during different periods of life, such as early adulthood versus middle age, or whether their interaction varies across an individual’s lifespan. A third and final limitation pertains to the characteristics of the sample. Despite efforts to include a diverse range of respondents, the majority of the sample identified as female university students from Europe, potentially limiting the generalizability of the results. For instance, self-concealment overlaps with emotional control, a practice valued and often expected in many Asian cultures (Liao et al., 2005). Therefore, administering the same survey within the context of Asian culture may yield slightly different findings. Additionally, due to a small number of individuals identifying as non-binary, they had to be excluded from the analysis. Future research should investigate how self-concealment and secrecy manifest in non-binary genders and across various cultural contexts. This is particularly important as much of the existing research on secrecy and self-concealment is based on binary genders and student populations, predominantly from the United States (Kawamura & Frost, 2004; Masuda et al., 2017; Vogel & Armstrong, 2010).

The findings of this study carry significant implications for both research and clinical practice. In terms of research, this study’s multifaceted approach to examining the relationship between self-concealment and secrecy provides a more comprehensive understanding of these constructs. Future research endeavors should consider adopting similar multifaceted perspectives to delve deeper into the intricate dynamics of secrecy and self-concealment. Moreover, longitudinal studies can shed light on how these relationships evolve over time and whether they differ across various life stages. In the realm of clinical practice, the results suggest that individuals exhibiting high self-concealment tendencies may benefit from interventions that address their attitudes toward secrecy and promote healthy coping strategies. Additionally, clinicians should be aware of the potential influence of self-concealment on clients’ secretive behaviors and encourage open communication to foster emotional well-being. Although high self-concealers have been found to have a more negative attitude toward therapy, they tend to seek therapy more frequently than low self-concealers (Kelly & Achter, 1995). Encouragingly, past research has demonstrated that self-concealment tendencies can decline over the course of therapy (Wild, 2005). Understanding the interplay between self-concealment and secrecy can aid mental health professionals in tailoring their therapeutic approaches to better serve their clients’ needs (Eǧeci & Gençöz, 2006; Meeks et al., 1998).

In conclusion, this study reaffirms the previously established link between high self-concealment and increased secrecy. Additionally, it sheds new light on the subject by revealing that high self-concealers tend to have a more favorable attitude toward secrecy. The importance of distinguishing between secrecy and self-concealment in future research is a key takeaway from this study, encouraging researchers to explore these constructs in diverse populations. Beyond academia, mental health professionals can apply these findings to better understand their clients’ tendencies toward self-concealment and secret-keeping, potentially enhancing the effectiveness of therapeutic interventions.

## Data Availability

The data that support the findings of this study are available from the corresponding author upon reasonable request. Preregistration: Not preregistered.
